# Forward‐planning intensity‐modulated radiotherapy technique for prostate cancer

**DOI:** 10.1120/jacmp.v8i4.2488

**Published:** 2007-11-05

**Authors:** Mohamed Metwaly, Awaad Mousa Awaad, El‐Sayed Mahmoud El‐Sayed, Abdel Sattar Mohamed Sallam

**Affiliations:** ^1^ Radiation Physics Department Faculty of Science Ain Shams University Cairo; ^2^ Radiotherapy Department Faculty of Science Ain Shams University Cairo Egypt; ^3^ Oncology and Hematology Hospital, Maadi Armed Forces Medical Compound Physics Department Faculty of Science Ain Shams University Cairo Egypt

**Keywords:** Intensity modulation, dynamic arc, prostate cancer, radiation dosimetry

## Abstract

In this study, we present an intensity‐modulated radiotherapy technique based on forward planning dose calculations to provide a concave dose distribution to the prostate and seminal vesicles by means of modified dynamic arc therapy (M‐DAT). Dynamic arcs (350 degrees) conforming to the beam's eye view of the prostate and seminal vesicles while shielding the rectum, combined with two lateral oblique conformal fields (15 degrees with respect to laterals) fitting the prostate only, were applied to deliver doses of 78 Gy and 61.23 Gy in 39 fractions to the prostate and seminal vesicles respectively. Dynamic wedges (45 degrees of thick end, anteriorly oriented) were used with conformal beams to adjust the dose homogeneity to the prostate, although in some cases, hard wedges (30 degrees of thick part, inferiorly oriented) were used with arcs to adjust the dose coverage to the seminal vesicles. The M‐DAT was applied to 10 patients in supine and 10 patients in prone positioning to determine the proper patient positioning for optimum protection of the rectum. The M‐DAT was compared with the simplified intensity‐modulated arc therapy (SIMAT) technique, composed of three phases of bilateral dynamic arcs. The mean rectal dose in M‐DAT for prone patients was 22.5±5.1 Gy; in M‐DAT and SIMAT for supine patients, it was 30.2±5.1 Gy and 39.4±6.0 Gy respectively. The doses to 15%, 25%, 35%, and 50% of the rectum volume in M‐DAT for prone patients were 44.5±10.2 Gy, 33.0±8.2 Gy, 25.3±6.4 Gy, and 16.3±5.6 Gy respectively. These values were lower than those in M‐DAT and in SIMAT for supine patients by 7.7%, 18.2%, 22.4%, and 28.5% and by 25.0%, 32.1%, 34.9%, and 41.9% of the prescribed dose (78 Gy) respectively. Ion chamber measurements showed good agreement of the calculated and measured isocentric dose (maximum deviation of 3.5%). Accuracy of the dose distribution calculation was evaluated by film dosimetry using a gamma index, allowing 3% dose variation and 4 mm distance to agreement as the individual acceptance criteria in prostate and seminal vesicle levels alike for all supine and prone patients. We found that fewer than 10% of the pixels in the dose distribution of the calculated area of 10×10−cm failed the acceptance criteria. These pixels were observed mainly in the low‐dose regions, particularly at the level of the seminal vesicles.

In conclusion, the single‐phase M‐DAT technique with patients in the prone position was found to provide the intended coverage of the prescribed doses to the prostate and seminal vesicles with improved protection for the rectum. Accordingly, M‐DAT has replaced non‐modulated conformal radiotherapy or SIMAT as the standard treatment for prostate cancer in our department.

PACS number: 87.53.Tf

## I. INTRODUCTION

Radiotherapy is one of the radical treatment options for early‐stage localized prostate cancer. An increase of radiation dose to the prostate is expected to increase local control, but is also associated with a dramatically increased risk of rectal complications.^(^ ^1^ ^,^ ^2^ ^)^ As compared with conventional radiotherapy, advanced radiotherapy techniques are designed to achieve prostate dose escalation while reducing side effects. Three‐dimensional conformal radiation therapy (3D‐CRT) facilitates prostate dose escalation and achieves higher disease‐free survival,^(^ ^3^ ^,^ ^4^ ^)^ comparable or lower acute toxicity,^(^ ^4^ ^–^ ^6^ ^)^ and lower late morbidity.^(^ ^6^ ^,^ ^7^ ^)^ The optimized 3D‐CRT plan can be achieved by proper choice of the number and orientations of conformal beams.^(^ ^8^ ^–^ ^12^ ^)^ None of the conformal treatment techniques studied proved to be an improvement with respect to sparing all the organs at risk.

In parallel to 3D‐CRT, arcs have been used as an effective alternative technique for prostate cancer radiotherapy. Based on forward‐planning dose calculation, arc therapy can be classified into three categories:

- conventional or standard arc therapy (SAT),
- conformal arc radiotherapy (CAT),
- and dynamic arc therapy (DAT).

In the SAT technique, the fixed collimator jaws define the field aperture during rotation. In CAT, the field aperture shape is designed to conform to the average planning target volume (PTV) projection shapes (beam eye views) during beam rotation by a fixed block or a multileaf collimator (MLC) shape. The main difference between CAT and DAT is that, in DAT, the MLC is moving to conform to the real beam eye views of the PTV at various gantry angles.

Comparisons of SAT and fixed fields showed that bilateral SAT resulted in a lower dose to the posterior rectal wall^(^ ^9^ ^,^ ^13^ ^,^ ^14^ ^)^ and that 360‐degree SAT resulted in better clinical (survival) outcome than did bilateral SAT.^(15)^ Comparisons of SAT and CAT showed that bilateral 120‐degree CAT significantly improved the dose distribution as compared with bilateral 120‐degree SAT.^(^ ^16^ ^,^ ^17^ ^)^ Three non‐coplanar 360‐degree CAT represented an improvement in the protection of rectum, bladder, and penile base, and consequently side effects were reduced despite high doses to the prostate and seminal vesicles.^(18)^

On the other hand, simplified intensity‐modulated arc therapy (SIMAT) appeared as a new modality of DAT for prostate cancer radiotherapy.^(19)^ The SIMAT technique is based on rotational fields of dynamically changing MLC apertures to conform to the PTV during beam rotation. These arcs are combined with others that conform to the PTV but that shield adjacent organs at risk. The three‐phase SIMAT technique was introduced to use forward‐planning dose calculation to produce the desired concave dose distribution that conforms well to the target (prostate only or prostate plus seminal vesicles), with sparing of rectum.^(^ ^19^ ^,^ ^20^ ^)^ The aim is the same as that for intensity‐modulated radiotherapy (IMRT) and intensity‐modulated arc therapy (IMAT), which are advanced techniques of 3D‐CRT and DAT respectively, in which the dose calculations are based on inverse planning.

In the present work, we are proposing a combination of SIMAT and 3D‐CRT to achieve the desired concave dose distribution to the prostate plus seminal vesicles. The technique is composed of full arcs that fit to the prostate plus seminal vesicles and that shield the rectum, combined with two lateral posterior oblique wedged conformal fields of low weighting that fit to the prostate only. The main difference between our technique and SIMAT is that the resulting concave dose distribution is produced by a combination of static conformal fields with dynamic conformal arcs in a single‐phase treatment. This technique has been termed “modified dynamic arc therapy” (M‐DAT).

The principal goal of M‐DAT is to provide higher doses to the prostate and seminal vesicles with maximal protection to the rectum. This approach minimizes the probability of undesirable hot spots or localized high‐dose regions in the subcutaneous tissues that would take place in cases using 3D‐CRT and IMRT. Also in the M‐DAT technique, the MLC shape of dynamic arcs can be designed automatically to fit to the prostate plus seminal vesicles and to shield the rectum at all gantry angles, making plan creation much easier. Moreover, the resulting dynamic MLC shape can be inspected before treatment simply by observing the beam eye views in the 3D planning system, which is not possible in cases of IMRT and IMAT.

We compared the M‐DAT technique with SIMAT to determine if M‐DAT provided advantages in sparing of rectum, bladder, and femoral heads, with similar or better coverage for the prescribed doses to prostate and seminal vesicles. The accuracy of the dose calculation algorithm of our planning system for M‐DAT had been determined by comparison with measurements.

Because we are focusing on a detailed presentation of M‐DAT technique and its comparison with SIMAT, a comparison of M‐DAT and IMRT plans is deferred to another publication.

## II. METHODS

### A. Computed tomography and magnetic resonance imaging scans and volume definition

We preformed computed tomography (CT) and magnetic resonance imaging (MRI) scans with a slice spacing of 3 mm for 20 patients of interest. The first 10 patients were in supine position; the others were in prone position. The scan borders were taken through the region from the lower end of the sacroiliac joint to the penile urethra plus 1 cm inferiorly and superiorly.

The CT and MRI images were transferred electronically to our Eclipse 3D planning system (version 7.3.10: Varian Medical Systems, Palo Alto, CA), in which image fusion was performed. Vacuum cushions (RepoVac‐type: Sinmed Radiotherapy Products, Reeuwijk, Netherlands) were used for patient immobilization and a Varian 23 EX linear accelerator with an 80‐leaf (40‐pair) MLC with a 1 cm width at the isocenter (software version 6.8.08: Varian Medical Systems) was used for treatment delivery.

For the purposes of the present study, the clinical target volume (CTV) was divided into two parts: prostate only (PO) and seminal vesicles (SV). Together, the volumes of the prostate and the seminal vesicles were the CTV. The margins for each subvolume were generated according to patient positioning reproducibility and to the PO displacement ranges in the various directions. For patients in the supine position, the most common directions of PO displacement are the anterior–posterior and superior–inferior, which are significantly larger than any left–right movement.^(^ ^21^ ^–^ ^28^ ^)^ Accordingly, we chose margins of 10 mm in the superior, inferior, and anterior directions, and 7 mm in the left–right direction. No margin was introduced between the PO and SV. In the posterior direction, the margin was taken as 6 mm to reduce rectal complications.^(29)^ These margins were increased by 3 mm in all directions to consider the potential for breathing that causes further prostate movement for patients in the prone position. These patients were educated for shallow breathing actions during CT or MRI scans and treatment delivery.^(30)^

The resulting planning target volumes for the PO, SV, and CTV were designated as the PPO, PSV, and PTV, respectively. The rectum (RC), bladder (BL), left and right femoral heads and necks (LF, RF), PO, and SV were contoured on MRI images, reviewed in CT images, and verified by the same radiation oncologist.

The RC was taken through the region from the sigmoid colon superiorly to the anal canal inferiorly. The outer longitudinal layers of muscles in each transversal section were included in this region. Instead of the RC, a reduced rectum volume was delineated to be shielded in dynamic arcs so as to minimize the effect of the MLC penumbra on the PPO coverage, with adequate RC shield. This rectum volume was delineated to be contracted away from the target by 6 – 8 mm or 8 – 10 mm for patients in the supine or prone position respectively. Because this volume is defined only for planning purpose, with no role in plan evaluation, we called it the “visual rectum volume” (VRV).

### B. Dose prescription

For SIMAT plans, the prescribed doses in phases I, II, and III were 54 Gy in 27 fractions, 12 Gy in 6 fractions, and 12 Gy in 6 fractions respectively.^(20)^ For M‐DAT plans, the prescribed doses for the PPO and PSV were 78 Gy and 61.23 Gy in 39 fractions. The prescribed dose to the PSV is equivalent to 54 Gy in 27 fractions as calculated using a computerized version of Orton's time–dose fractionation tables^(31)^ (contributed by Maria A. Czerminska, Radiology, University of Illinois, Chicago, IL).

### C. SIMAT technique

Three‐phase SIMAT plans were created and calculated using CT images of the 10 supine patients. In phase I, the bilateral arcs covered the PSV; however, they covered the PPO in the other two phases. In phases I and II, non‐shielded arcs of ranges 45 degrees – 145 degrees for left arcs and 215 degrees – 315 degrees for right arcs were used. In phase III, shielded RC arcs of ranges 90 degrees – 180 degrees for left arcs and 180 degrees – 270 degrees for right arcs were used. A margin of 5 mm between field edges and the PTV was taken in all directions.^(20)^ The SIMAT plan was produced by the sum of the three phases using the “plan sum” option of the Eclipse planning system.

### D. M‐DAT technique

**Figure 1:**
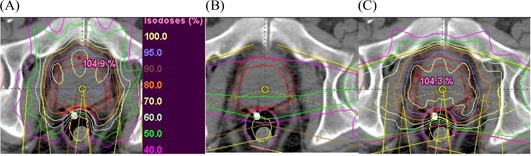
Fields arrangement and isodose distribution for (A) the two shielded arcs, (B) bilateral conformal beams, and (C) their combination.

Two arcs covering 350 degrees (one clockwise and the other counterclockwise) were generated automatically by the planning system to fit the PTV and shield the VRV. The two conformal fields of symmetrical angles with respect to laterals were assigned to cover the PPO. The target volume dose uniformity is produced by the combination of a high‐dose volume yielded anteriorly by arcs with that yielded posteriorly by conformal fields as shown in Fig. 1. The margins of the MLC aperture to the PTVs for all fields were taken 5 mm in all directions, except superiorly for the PSV (for arcs) and inferiorly for the PPO (for arcs and static fields), where the margin was 8 mm. This approach was adequate to cover 100% of the PTVs with 95% of the prescribed doses. Three gantry angles [0 degrees (A0), 15 degrees (A15), and 30 degrees (A30)] downward to laterals for supine patients and upward to laterals for prone patients were tested in a trial‐and‐error fashion to find the optimum conformal field angles. We thought that applying wedges (anteriorly oriented thick ends) to the two conformal fields, with lowering of their weighting, might help in sparing the femoral heads. For optimization purposes, a set of enhanced dynamic wedges of angles 0 degrees, 30 degrees, 45 degrees, and 60 degrees (W0 to W60) were tested in a trial‐and‐error fashion to find the optimum gantry angle. In some cases, the dose to the PSV was adjusted by a hard wedge of angle 30 degrees with shielded arcs such that its thin part was oriented superiorly in the PSV direction.

### E. Plan evaluation and comparisons

The dose–volume histograms (DVHs) for the PTVs and organs at risk were used for plan evaluation and comparisons. All plans were normalized based on the DVHs to ensure that 95% of the PTV received 100% of the prescribed dose. The dose inhomogeneity (DI) in the PTV was defined as $(D_{5}-D_{95})/D_{\text{mean}}$.^(32)^ (Table 1 shows the definitions of $D_{5},D_{95}$, and $D_{\text{mean}}$.)

**Table 1:** Dose and volume comparison metrics

Parameter	Definition
$D_{\text{max}},D_{\text{min}},D_{\text{mean}},D_{\text{modal}},D_{\text{median}}$	The maximum, minimum, mean, modal, and median doses to the volume of interest.
$V_{84}$	The percentage of the planning target volume covered by 84 Gy.
$V_{D\text{min}}$	The percentage volumes of the minimum doses.
$D_{5},D_{15},D_{25},D_{35},D_{50},D_{95}$	The doses to 5%, 15%, 25%, 35%, 50%, and 95% of the volume of interest.
$V_{52},V_{50},V_{60}$	The parentage of any volume covered by 52, 50, or 60 Gy.

The M‐DAT plans were considered acceptable if they satisfied the dose guidelines designed by the Radiation Therapy Oncology Group (RTOG) for patients being treated for localized prostate cancer under RTOG protocol 0126.^(33)^ According to the RTOG guidelines, rectal criteria require that no more than 15%, 25%, 35%, and 50% of the rectum volume should receive more than 75 Gy, 70 Gy, 65 Gy, and 60 Gy respectively. For the bladder, the guidelines require that no more than 15%, 25%, 35%, and 50% of the bladder volume should receive more than 80 Gy, 75 Gy, 70 Gy, and 65 Gy respectively. In addition, no more than 2% of the PTV is to receive more than 84.7 Gy and no less than 98% is to be covered by the prescribed dose (78 Gy for the PPO and 54 Gy for the PSV). The femoral head doses are not mentioned in RTOG 0126. We applied the criteria for acceptable femoral head doses set out in report 62 from the International Commission on Radiation Units and Measurements (ICRU),^(34)^ in which the volume that is covered by 52 Gy or more should be minimized.

Table 1 lists the definitions of doses and volumes used for optimization and comparison. The mean DVHs were taken separately for supine and prone patients. The M‐DAT was applied for supine and prone patients; the SIMAT was calculated for supine patients only. Results were then compared.

### F. Plan verification

Ion chamber and film dosimetry were used to verify the doses calculated by the Eclipse planning system for the M‐DAT plans. The plans for all patients were exported in Eclipse to the CT images of a cylindrical phantom (PTW T9193: PTW, Freiburg, Germany) and calculated for comparison with ion chamber measurements. The plans of all supine and prone patients were also exported to the CT images of an Alderson Rando anthropomorphic phantom (PTW) and calculated for comparison with film measurements.

A pinpoint ion chamber (PTW 31006, 0.015 cm^3^: PTW) located in the central axis of the cylindrical phantom was used to verify the calculated isocenter dose. The measurements were performed at levels corresponding to the central axial levels of prostate and seminal vesicles in the phantom and were then compared with the calculated doses at these points.

Before film calibration, the machine output of the 6‐MV photon beam was adjusted such that the beam output corresponded to 1 cGy per monitor unit (MU) at $d_{\text{max}}$ for a 10×10−cm field at a source‐to‐surface distance (SSD) of 100 cm. Five Kodak extended dose range (EDR2) films (for calibration) were individually inserted in the spacing of the central slabs of a 30×30×30 cm Solid Water (Gammex rmi, Middleton, WI) phantom (PTW‐29672: PTW). The phantom was positioned to place the film in the beam central axis plane at a 90‐degree gantry angle with a field size of 10×10 cm and a SSD of 100 cm. The films were exposed to the 6‐MV photon beam at five different MU values (50, 200, 400, 700, and 1000 MUs) and were processed 1 hour after irradiation^(35)^ by an automatic processing machine (AFP Imaging, Elmsford, NY) and scanned by a Vidar‐16 scanner (Vidar Systems, Herndon, VA). The relationship between the depth and the optical density (OD) for each calibration film was determined by PTW Film Analysis software (version 1.3: PTW) and exported to an Excel (Microsoft, Redmond, WA) spreadsheet.

To determine the relationship between the dose and the OD, the percentage depth dose data of the 6‐MV photon beam for the 10×10−cm field size at a SSD of 100 cm was converted in the same Excel spreadsheet into 5 steps that corresponded to the MU values used during the film exposure. As a result, at all depths in each film, the correspondence between OD and dose could be determined. To reduce the variability of working conditions, all calibration and dosimetry measurements were performed in a single session.^(36)^

For M‐DAT plan verification, two films were inserted axially into the Alderson phantom at two levels located in the region of the prostate and seminal vesicles for each patient of interest. The PTW‐Varisoft 3.1 software was used to import and compare the dose distributions measured by film and calculated by Eclipse at the same levels. The criterion used to evaluate the accuracy of the Eclipse calculations was the gamma index,^(37)^ with individual acceptance criteria of 3% dose difference (DD) and 4 mm distance to agreement (DTA). A scanned and calculated area of 10×10 cm was found to be adequate for including the isodose lines above 50%. A quantitative analysis of the dose distribution comparison based on gamma reports (a report contains the total number of pixels evaluated and the percentage passed and failed) was performed to show the percentage of pixels in the distribution that exceeded the acceptance criteria (percentage failed pixels) for both the PO and the SV level for supine and prone patients.

## III. RESULTS AND DISCUSSION

### A. SIMAT plan

Our SIMAT planning results agreed with previously published data.^(20)^ With respect to the PPO coverage, the calculated $D_{\text{max}}$ and $D_{\text{mean}}$ indicated that our results in the present study are higher by only 2.44% and 2.28% respectively. Also, the values of $D_{\text{max}}$ in case of the RC and LF were lower only by 1.47% and 3.62% respectively. Our results indicated that the SIMAT plan could be more protective to the RC and LF than that previously published,^(20)^ because the $D_{\text{mean}}$ values were lower by 7.10% and 17.36% respectively. The large difference in the $D_{\text{mean}}$ for the LF could be attributable to the definition of that structure, because the LF is delineated as both head and neck in the present study, but in the previous publication,^(21)^ it was delineated as head only.

### B. M‐DAT plan optimization

**Figure 2:**
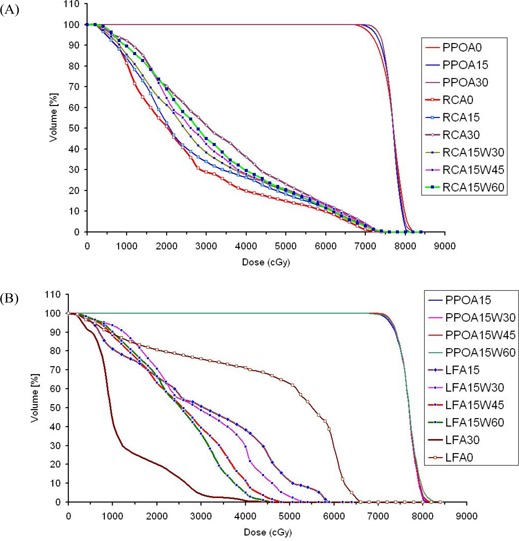
Dose–volume histograms of a supine patient for (A) planning prostate‐only (PPO) volume and rectum (RC) with gantry angles A0, A15, and A30, and RC with gantry angle A15 when various dynamic wedges (W30, W45, and W60) were used; and (B) head and neck of left femur (LF) with gantry angles A0, A15, and A30, and PPO and LF with gantry angle A15 when various dynamic wedges (W30, W45, and W60) were used.

**Figure 3:**
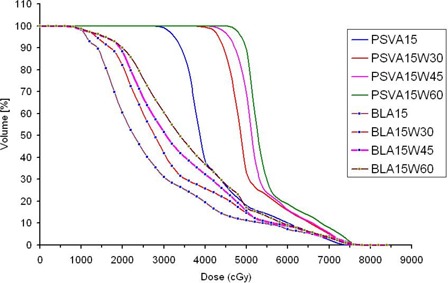
Dose–volume histograms of a supine patient for planning seminal vesicles–only (PSV) volume and bladder (BL) with gantry angle A15 when various dynamic wedges (W30, W45, and W60) were used.

Figs. 2 and 3 present examples of DVHs for all volumes of interest with three conformal field angles (A0, A15, and A30) and various dynamic wedge angles (W30, W45, and W60). Because of the symmetric field arrangement in the M‐DAT plan, the DVHs of the femoral heads are represented only by the LF. Fig. 2 demonstrates the effect on the DVHs of the PPO, RC, and LF of variation of the conformal field angles: as the angle increases, the RC dose increases and the LF dose decreases with minimal change in PPO coverage. Reasonably, as the two conformal fields moved posteriorly (downward for supine patients or upward for prone patients), their crossing volume in rectum increases [Fig. 2(A)] and their coverage of the femoral heads decreases [Fig. 2(B)]. Consequently, the angle A0 is optimum with respect to rectum protection, but worse with respect to femoral head protection as a result of the undesirable alignment of the two conformal fields with the two femoral heads at this angle. Therefore, A15 is the optimum gantry angle for the conformal fields.

To reduce the LF doses, enhanced dynamic wedges were used such that their thick ends were oriented anteriorly in both conformal fields. Using this adjustment, we were able to reduce the weighting of conformal fields, which significantly reduced the LF dose with the A15 gantry angle [Fig. 2(B)]. Further reduction of the dose to the LF was obtained with greater wedge angles with more reduction of the weighting of the conformal fields [Fig. 2(B)]. On the other hand, as Fig. 3 shows, the doses to the PSV and BL increased as the wedge angle increased with the same gantry angle because of enhancement of the dose contribution of the shielded arcs as a result of the reduction of the weighting of the conformal fields. For obese patients in the supine position particularly, such enhancement was not sufficient to cover the seminal vesicles with the prescribed dose. This situation is the result of the extra attenuation carried out by the abdominal fat strip that faced the PSV with shielded arcs. Consequently, a 30‐degree hard wedge (thin end oriented to the PSV) was used with shielded arcs to increase the dose to the PSV volume at the expense of dose to the PPO volume. The plan was then renormalized to achieve the desired dose coverage to both the PPO and PSV (100% of the PTVs covered by 95% of the prescribed dose). Table 2 presents typical parameters for the optimized M‐DAT plan.

**Table 2:** Optimum setup parameters^a^ of the modified dynamic arc therapy plan for the Varian 23 EX machine in the Varian in the International Electrotechnical Commission (IEC) scale

	VRV shielding	Gantry (degrees)	Collimator	Wedge	Weighting
Arc1	Yes	175–185 anticlockwise	0	Hard wedge 30 in (in 4 cases)	1.5–1.65
Arc2	Yes	185–175 clockwise	0	Hard wedge 30 in (in 4 cases)	1.5–1.65
LT	No	105	90	EDW 45 or 60 in	0.40–0.55
RT	No	255	90	EDW 45 or 60 out	0.40–0.55

a The normalization condition was 100% of the planning target volumes covered by 95% of the prescribed dose. VRV=visual rectum volume; ARC1=arc number 1; ARC2=arc number 2; LT=left oblique conformal field; EDW=enhanced dynamic wedge; RT=right oblique conformal field.

### C. Plan comparisons

Comparison of the dose distributions to the PPO and PSV indicated that SIMAT and M‐DAT for supine patients are similar except for the PO dose homogeneity (represented by the DI), which, as Table 3 shows, is better in SIMAT. Table 3 also shows that the PSV dose homogeneity in SIMAT is better than that in M‐DAT, but that the dose coverage (represented by the mean, modal, and median) was better in M‐DAT.

**Table 3:** Dose comparison of modified dynamic arc therapy (M‐DAT) and simplified intensity‐modulated arc therapy (SIMAT) plans concerning all regions of interest for 10 supine and 10 prone patients

	PPO [mean (±SD) Gy]		PSV [mean (±SD) Gy]		RC [mean (±SD) Gy]			BL [mean (±SD) Gy]			LF [mean (±SD) Gy]	
	SIMAT	M‐DAT	SIMAT	M‐DAT	SIMAT	M‐DAT		SIMAT	M‐DAT		SIMAT	M‐DAT
		Supine and prone		Supine and prone		Supine	Prone		Supine	Prone		Supine and prone
$D_{\text{min}}$	73.92	73.92	50.0	52.9	4.19	3.77	1.4	2.15	8.91	6.5	4.39	3.76
	(5.2)	(1.0)	(3.2)	(2.5)	(1.4)	(1.4)	(0.5)	(1.2)	(5.0)	(4.0)	(4.1)	(2.0)
$D_{\text{max}}$	81.00	83.10	78.04	80.05	70.65	70.51	78.8	76.37	79.37	78.8	57.48	56.80
	(0.8)	(0.2)	(1.2)	(1.5)	(8.2)	(7.3)	(3.4)	(0.9)	(2.6)	(3.2)	(14.2)	(4.2)
$D_{\text{mean}}$	77.88	77.88	54.79	58.9	39.36	30.22	22.5	25.85	34.05	32.8	27.16	27.41
	(0.2)	(0.4)	(1.6)	(3.3)	(6.0)	(5.1)	(5.1)	(5.1)	(8.5)	(8.0)	(5.6)	(6.1)
$D_{\text{median}}$	78.00	78.00	52.01	56.5	40.84	26.99	16.9	20.36	31.52	28.2	26.28	27.33
	(0.2)	(0.4)	(0.7)	(3.7)	(8.1)	(5.8)	(6.0)	(12.4)	(9.4)	(8.1)	(11.4)	(8.4)
$D_{\text{modal}}$	78.42	78.42	52.05	57.48	54.73	20.94	18.9	13.53	23.62	20.7	24.97	29.38
	(1.3)	(1.3)	(0.4)	(1.0)	(10.2)	(7.6)	(4.0)	(5.6)	(8.8)	(7.2)	(5.6)	(9.4)
DI^a^	0.077	0.089	0.33	0.42								
	(0.01)	(0.01)	(0.05)	(0.02)								
	(%)	(%)	(%)	(%)								
$V_{D\text{min}}$	99.8	99.8	99.5	98.7								
	(0.1)	(0.2)	(0.3)	(0.1)								

a Dose inhomogeneity $=(D_{5}-D_{95})/D_{\text{mean}}$. Table 1 gives the definitions of $D_{5},D_{95}$, and $D_{\text{mean}}$.

PPO=planning prostate‐only volume; SD=standard deviation; PSV=planning seminal vesicles–only volume; RC=rectum; BL=bladder; LF=left femoral head and neck.

**Figure 4:**
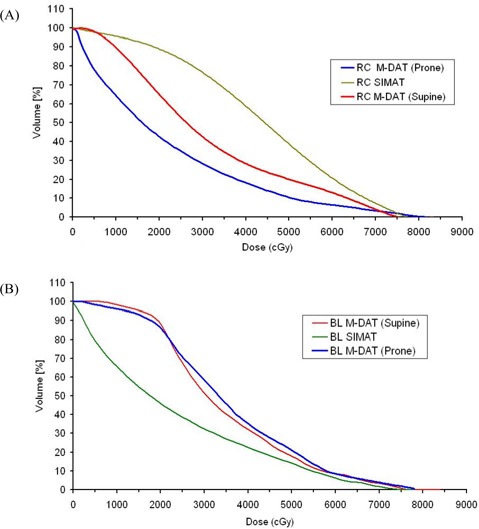
Plot of mean dose–volume histograms of 10 supine and 10 prone patients for (A) rectum and (B) bladder with simplified intensity modulated arc therapy (SIMAT) and modified dynamic arc therapy (M‐DAT). RC=rectum; BL=bladder.

We noted no significant variations in the PPO and PSV dose distributions between M‐DAT plans for supine and prone patients. The values of $D_{\text{max}}$ in Table 3 indicated that no volume of the PPO is covered by 84.7 Gy $\left(D_{\text{max}}<84.7\right)$. This result respects the RTOG 0126 criteria for maximum dose coverage to the target. Rectum protection in SIMAT is worse than that in M‐DAT for the supine position, but M‐DAT in the prone position provides the best rectum protection, as shown in (Fig 4A). This result is attributable to the enlargement of rectum size posteriorly in the prone position, which results in displacement of most of the rectal wall away from the treatment volume as noted in comparisons of the rectum shape in CT images of all supine and prone patients under study.

Table 3 indicates that all statistical parameters of rectum dose distribution for prone patients are superior with respect to rectum protection except for the $D_{\text{max}}$ as Fig. 2 also shows. This result is attributable to the extra margin (by 3 mm) taken in all directions to the PO and SV in the construction of the PPO and PSV to consider the potential for breathing to influence prostate movement for patients in the prone position.

On the other hand, SIMAT is better than M‐DAT in both the supine and the prone position with respect to low‐dose (<55 Gy) protection to the bladder [Fig. 4(B)]. This finding is a consequence of the higher delivered doses to the PSV volume in M‐DAT (Table 3). Fig. 4(B) also shows that bladder doses are not influenced by patient positioning with the M‐DAT technique.

Table 4 demonstrates the quantitative analysis of the DVHs based on our comparison criteria mentioned earlier and presented in Table 1. Accordingly, the doses to 15%, 25%, 35%, and 50% of the rectum volume with M‐DAT in the supine position are 7.7%, 18.2%, 22.4%, and 28.5% lower than those with SIMAT, but higher than those with M‐DAT in the prone position by 17.3%, 13.8%, 12.4%, and 13.5% of the prescribed dose respectively.

**Table 4:** The dose–volume histogram quantitative comparisons of modified dynamic arc therapy (M‐DAT) and simplified intensity modulated arc therapy (SIMAT) plans concerning organs at risk for 10 supine and 10 prone patients

	RC [mean (±SD) Gy]			BL [mean (±SD) Gy]			LF [mean (±SD) Gy]	
	SIMAT	M‐DAT		SIMAT	M‐DAT		SIMAT	M‐DAT
		Supine	Prone		Supine	Prone		Supine and prone
$D_{15\%}$	64.00	58.00	44.50	49.00	50.00	54.50	38.00	37.10
	(12.3)	(11.5)	(10.2)	(17.3.0)	(15.2.5)	(11.4)	(16.6)	(14.3)
$D_{25\%}$	58.00	43.80	33.00	37.50	45.00	47.00	32.00	32.80
	(5.0)	(6.2)	(8.2)	(15.4)	(12.2)	(15.2)	(15.0)	(10.5)
$D_{35\%}$	52.50	35.00	25.30	28.50	38.00	39.50	31.50	32.00
	(6.4)	(8.3)	(6.4)	(12.4)	(10.8)	(11.5)	(19.4)	(16.8)
$D_{50\%}$	49.00	26.80	16.30	18.00	30.50	33.50	26.10	26.10
	(7.0)	(7.5)	(5.6)	(10.5)	(14.6)	(12.2)	(8.5)	(9.7)
	(%)	(%)	(%)	(%)	(%)	(%)		
$V_{50}$	38.0	20.0	10.0	23.0	15.0	18.0	0.0	0.0
	(5.2)	(3.2)	(5.3)	(8.2)	(5.4)	(5.6)		
$V_{60}$	22.0	13.0	7.0	5.0	9.0	9.0	0.0	0.0
	(9.2)	(6.2)	(5.2)	(2.2)	(5.2)	(4.2)		

RC=rectum; SD=standard deviation; BL=bladder; LF=left femoral head and neck.

The percentage rectum volumes covered by 50 Gy and 60 Gy with M‐DAT in the supine position are 18% and 9% lower than those with SIMAT, but they are higher than those with M‐DAT in the prone position by 10.0% and 6% respectively.

Tables 3 and 4 indicate that the LF volume covered by doses exceeding 52 Gy are negligible in the SIMAT and M‐DAT techniques alike. Consequently, they are acceptable with respect to LF protection under ICRU criteria.^(34)^

Follow‐up of all patients showed clinical regression of prostatic tumor as evidenced by CT imaging and measurement of blood levels of prostate‐specific antigen at 3‐month intervals. All patients were weaned off total androgen blockade during a period of 6 – 12 months after radical radiotherapy. Acute cystitis during treatment was tolerable and was treated symptomatically Rectal and bladder complications were minimal after 1 year of follow‐up. From our results and those of pervious studies,^(^ ^38^ ^–^ ^40^ ^)^ predictable late rectal bleeding after M‐DAT in the prone position is reasonably lower than that with either SIMAT or with M‐DAT in the supine position. Earlier studies had shown that late rectal toxicity is significantly correlated with the absolute and percentage volume of rectum receiving all ranges of dose.

### D. Plan verification

**Figure 5:**
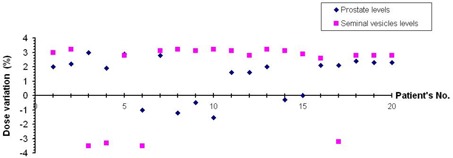
The percentage variation of the calculated and measured doses in the levels of prostate and seminal vesicles for the 10 supine (1 – 10) and 10 prone (11 – 20) patients, including 4 cases (3, 4, 6, 17) of wedged arcs.

The absolute dose measurements performed at the isocentric axis of the cylindrical phantom indicated that dose values at the chosen levels representing prostate and seminal vesicles are in agreement with the calculated doses at the same points. Fig. 5 shows the percentage variation of the calculated and measured doses at the levels of prostate and seminal vesicles for 10 supine (1 – 10) and 10 prone (11 – 20) patients including 4 cases (3, 4, 6, 17) of wedged arcs. At the prostate level, the mean variation between measured and calculated doses and the maximum discrepancy were 1.7% and 3% respectively. However, at the level of the seminal vesicles, they were 2.6% and −3.5% respectively. Maximum discrepancies are noted when wedged arcs are applied.

**Figure 6:**
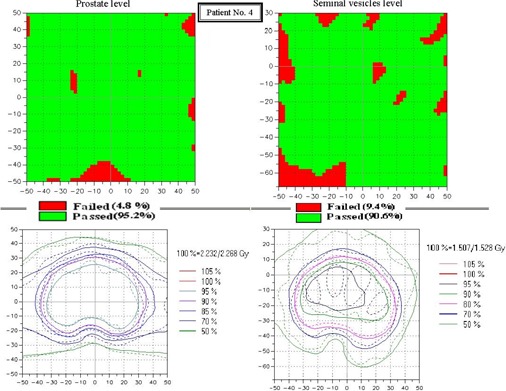
An example of gamma distribution (upper parts) and the measured and calculated modified dynamic arc therapy (M‐DAT) dose distributions (lower parts) for prostate (left side) and seminal vesicle (right side) levels for a 10×10−cm scanned and calculated area. (Scale of the coordinate axes is 10 mm.) The green areas indicate regions where pixels passed the gamma acceptance criteria (3% dose difference and 4 mm distance to agreement); the red, regions where pixels failed. The continuous and dashed lines represent the measured and calculated dose distributions respectively.

**Figure 7:**
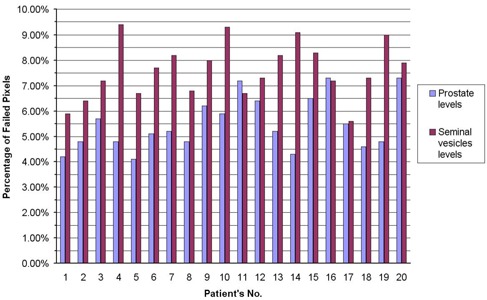
Percentage of failed pixels in a modified dynamic arc therapy (M‐DAT) dose distribution as compared with the gamma acceptance criteria (3% dose difference and 4 mm distance to agreement) for prostate and seminal vesicle levels of 10 supine (1 – 10) and 10 prone (11 – 20) patients, including 4 cases (3, 4, 6, 17) of wedged arcs.

Fig. 6 shows an example of gamma distributions and measured and calculated dose distributions for the prostate and seminal vesicle levels. Notably, the main region of dose distribution that failed the acceptance criteria (3% DD and 4 mm DTA) was located in the low‐dose regions, particularly at the level of the seminal vesicles. Fig. 7 shows the percentage of failed pixels in the distribution for both the prostate and the seminal vesicle levels for the supine (1 – 10) and the prone (11 – 20) patients. It is easy to see that, at the prostate level, the dose distribution is more accurately calculated than at the seminal vesicle level. In all cases, fewer than 10% of the pixels failed the acceptance criteria even with wedged arcs (patients 3, 4, 6, and 17).

## IV. CONCLUSION

The optimized single‐phase M‐DAT technique produced distinctly favorable dose distributions for coverage of the prostate and seminal vesicles and for protection of rectum and femoral heads. In addition, the desired concave dose distribution is obtained with a single‐phase arc therapy technique based on forward planning dose calculation. Compared with SIMAT treatments, M‐DAT treatments produce less bladder protection in the lower doses regions, but with consistently greater sparing of rectum in regions of higher and lower dose. When treatment of the prostate only is required, the technique documented here can be applied by removing the seminal vesicles from the dynamic arc apertures. In general, the M‐DAT technique combines the advantages of arc therapy in the treatment of the central lesion with maximum protection for the long organ at risk near the lesion of interest. This technique may be investigated for spinal cord protection in the treatment of esophageal, para‐aortic, and head‐and‐neck lesions.

## ACKNOWLEDGMENT

We thank our consultants and colleagues in the departments of Radiation Physics and Radiotherapy in Maadi Armed Forces Medical Compound, and especially Prof. Dr. Mossad Hegazy for his radiobiologic background support.

## Supporting information

Supplementary Material Files

Supplementary Material Files
