# Supplementary material for: Forward‐planning intensity‐modulated radiotherapy technique for prostate cancer
Source: J Appl Clin Med Phys. 2007 Nov 5;8(4):114–28. doi: 10.1120/jacmp.v8i4.2488 (PMC5722620; doi:10.1120/jacmp.v8i4.2488)
Supplement: Supplementary file 1 — Supplementary Material Files [file ACM2-8-114-s001.doc]

**Forward Planning Intensity Modulated Radiotherapy Technique for Prostate Cancer**

**M.Metwaly^1^, A.M.Awad^2^, E.M. El-Sayed^3^, A.M. Sallam^3^**

*^1^ Radiation Physics Department, Oncology and Hematology Hospital, Maadi Armed Forces Medical Compound, Cairo, Egypt.*

*^2^ Radiotherapy Department, Oncology and Hematology Hospital, Maadi Armed Forces Medical Compound, Cairo, Egypt.*

*^3^ Physics Department, Faculty of science, Ain Shams University, Cairo, Egypt.*

**Corresponding author**: Mohamed Metwaly, Department of *Radiation Physics*, *Oncology and Hematology Hospital, Maadi Armed Forces Medical Compound, Cairo, Egypt.*

Mobile: (+20) 123614656; E-mail: mmettelk@yahoo.com

**Forward Planning Intensity Modulated Radiotherapy Technique for Prostate Cancer**

**ABSTRACT**

In this study an intensity modulated radiotherapy technique based on forward planning dose calculations is presented to provide a concave dose distribution to the prostate and seminal vesicles by means of Modified Dynamic Arc therapy (M-DAT). Dynamic arcs (350°) conforming to the beam's eye view of the prostate and seminal vesicles while shielding the rectum combined with two laterals oblique conformal fields (15^o^ with respect to laterals) fitting the prostate only were applied to deliver doses of 78 Gy and 61.23 Gy in 39 fractions to the prostate and seminal vesicles respectively. Dynamic wedges (45° of thick end anteriorly oriented) were employed with conformal beams to adjust the dose homogeneity to the prostate, although in some cases hard wedges (30° of thick part inferiorly oriented) were used with arcs to adjust the dose coverage to the seminal vesicles. M-DAT was applied to 10 patients in supine and 10 others in prone positioning to determine the proper patient positioning for optimum protection of the rectum. M-DAT was compared with the simplified intensity modulated arc therapy (SIMAT) technique composed of three phases of bilateral dynamic arcs. The mean rectal dose in M-DAT for prone patients was (22.5 ± 5.1) Gy while it was (30.2 ± 5.1) Gy and (39.4 ± 6.0) Gy in M-DAT and SIMAT for supine patients, respectively. The doses to 15%, 25%, 35% and 50% of the rectum volume in M-DAT for prone patients were (44.5 ± 10.2) Gy, (33.0 ± 8.2) Gy, (25.3 ± 6.4) Gy and (16.3 ± 5.6) Gy, respectively. These values were lower than that in M-DAT and SIMAT for supine patients by (7.7 %, 18.2 %, 22.4 % and 28.5 %) and (25.0 %, 32.1 %, 34.9 % and 41.9 %) of the prescribed dose (78 Gy), respectively. Ion chamber measurements showed good agreement of the calculated and measured isocentric dose (maximum deviation of 3.5%). Accuracy of the dose distribution calculation was evaluated by film dosimetry using a gamma index, allowing 3% dose variation and 4 mm distance to agreement as the individual acceptance criteria, in both prostate and seminal vesicles levels for all supine and prone patients. It was found that less than 10 % of the pixels in the dose distribution of the calculated area of 10 x 10 cm^2^ failed the acceptance criteria. These pixels were observed mainly in the low-dose regions, particularly at the levels of seminal vesicles.

In conclusion, the single phase M-DAT technique with patients in prone positioning was found to provide the intended coverage of the prescribed doses to the prostate and seminal vesicles with improved rectum protection. Accordingly, M-DAT has replaced non-modulated conformal radiotherapy or SIMAT as the standard treatment for prostate cancer in our department.

Key words: Intensity modulation, Dynamic arc, prostate cancer, radiation dosimetry

PACS number: 87.53.Tf

**I. INTRODUCTION**

Radiotherapy is one of the radical treatment options for early stage localized prostate cancer. An increase of radiation dose to the prostate is expected to increase the local control, but is also associated with a dramatically increased chance of developing rectal complications [1, 2]. Advanced radiotherapy techniques are designed to achieve prostate dose escalation, while reducing side effects compared with conventional radiotherapy. Three-dimensional conformal radiation therapy (3D-CRT) facilitates prostate dose escalation, achieves higher disease-free survival [3, 4], comparable or lower acute toxicity [4, 5, 6] and lower late morbidity [6, 7]. The optimized 3D-CRT plan can be achieved by a proper choice of number and orientations of conformal beams [8-12]. None of the conformal treatment techniques studied proved to be an improvement with respect to sparing all the organs at risk.

In parallel to 3D-CRT, arcs were used as an effective alternative technique for prostate cancer radiotherapy. Based on forward planning dose calculation, one may classify arc therapy into three categories: conventional or standard arc therapy (SAT), conformal arc radiotherapy (CAT), and dynamic arc therapy (DAT). SAT is a technique in which the fixed collimator jaws define the field aperture during rotation. In CAT the field aperture shape is designed to conform to the average planning target volume (PTV) projection shapes (beam eye views) during beam rotation by a fixed block or a multileaf collimator (MLC) shape. The main difference between CAT and DAT is that in DAT the MLC is moving to conform to the real beam eye views of PTV at the different gantry angles.

Comparisons of SAT and fixed fields showed that bilateral SAT gave a lower dose to posterior rectal wall [9, 13 and 14] and 360^o^ SAT represented better clinical (survival) outcome compared with bilateral SAT [15]. Comparisons of SAT and CAT showed that bilateral 120° CAT significantly improved the dose distribution compared to bilateral 120° SAT [16, 17]. Three non-coplanar 360° CAT represented an improvement in protection of rectum, bladder, and penile base and consequently side effects were reduced despite high doses to the prostate and seminal vesicles [18].

On the other hand a simplified intensity modulated arc therapy (SIMAT) appeared as a new modality of DAT for prostate cancer radiotherapy [19]. SIMAT is based on rotational fields of dynamically changing MLC apertures to conform to the PTV during beam rotation. These arcs are combined to others that conform to the PTV but shield adjacent organs at risk (OAR). The three phase SIMAT technique was introduced to produce the desired concave dose distribution that conforms well to the target (prostate only or prostate plus seminal vesicles) with sparing of rectum using forward planning dose calculation [19, 20]. This is the same aim as intensity modulated radiotherapy (IMRT) and intensity modulated arc therapy (IMAT) which are advanced techniques of 3D-CRT and DAT, respectively, in which the dose calculations are based on inverse planning.

In this work we are proposing a combination of SIMAT and 3D-CRT to achieve the desired concave dose distribution to the prostate plus seminal vesicles. The technique is composed of full arcs fitting the prostate plus seminal vesicles while shielding the rectum combined with two lateral posterior oblique wedged conformal fields of low weighting fitting the prostate only. The main difference between our technique and SIMAT is that the resulting concave dose distribution is produced by a combination of static conformal fields with dynamic conformal arcs in a single phase treatment. This technique has been termed “Modified Dynamic Arc therapy” (M-DAT).

The principal goal of M-DAT is to provide higher doses to the prostate and seminal vesicles with maximal protection to the rectum. This would minimize the probability of the undesirable hot spots or localized high dose regions in the subcutaneous tissues that would take place in cases of 3D-CRT and IMRT. Also in this technique the MLC shape of dynamic arcs can be designed automatically to fit the prostate plus seminal vesicles and shield the rectum at all gantry angles which makes the plan creation much easier. Moreover, the resulted dynamic MLC shape can be inspected before treatment simply by observing the beam eye views in the 3D planning system which is not possible in cases of IMRT and IMAT.

The M-DAT technique was compared with SIMAT to determine if M-DAT provided advantages in sparing of rectum, bladder and femoral heads with similar or better coverage of the prescribed doses to prostate and seminal vesicles. The accuracy of the dose calculation algorithm of our planning system for M-DAT had been determined by comparison with measurements.

Since we are focusing on the detailed presentation of M-DAT technique and comparison with SIMAT, it is preferred to put off the comparison of M-DAT and IMRT plans to another publication.

**II. METHODS**

**A. CT and MRI scans and volumes definition**

CT and MRI scans with a slice spacing of 3 mm were preformed for 20 patients of interest. The first 10 patients were in supine positioning while the others were in prone positioning. The scan borders were taken through the region from the lower end of the sacroiliac joint down to the penile urethra plus 1 cm inferiorly and superiorly.

The CT and MRI images were transferred electronically to our Eclipse 3D planning system version 7.3.10 (Varian Medical Systems inc., Palo Alto, USA), in which image fusion had been performed. Vacuum cushions (RepoVac- type, Sinmed Radiotherapy Products, The Netherlands) were used for patient immobilization and a Varian 23 EX machine with an 80 leaf (40 pairs) MLC of 1cm width at the isocenter (software version 6.8.08, Varian Medical Systems inc., Palo Alto, USA) was used for the treatment session delivery.

For the purpose of this study, the clinical target volume was divided into two parts: prostate only (PO) and seminal vesicles (SV). The volume of prostate plus seminal vesicles was named CTV. The margins for each volume were generated according to patient positioning reproducibility and the PO displacement ranges in the different directions. For patients in the supine positioning the most common directions of displacement of the PO are in anterior-posterior and superior-inferior direction, which are significantly larger than any left-right movement [21-28]. Accordingly, we chose margins of 10 mm in superior, inferior and anterior directions and 7 mm in the left-right direction. No margin was taken between the PO and SV. In the posterior direction the margin was taken as 6 mm to reduce rectal complication [29]. These margins were increased by 3 mm in all directions to consider the potential for breathing that causes further prostate movement for patients in prone positioning. These patients were educated for shallow breathing actions during CT or MRI scans and treatment delivery [30].

The resulting planning target volumes for the PO, SV and CTV were designated as the PPO, PSV and PTV, respectively. The rectum (RC), bladder (BL), left and right femoral heads and necks (LF and RF), PO and SV were contoured on MRI images, reviewed in CT images and verified by one radiation oncologist.

The rectum was taken through the region from the sigmoid colon superiorly to the anal canal inferiorly. The outer longitudinal layers of muscles in each transversal section were included in this region. A reduced rectum volume was delineated to be shielded in dynamic arcs instead of the RC to minimize the effect of the MLC penumbra on the PPO coverage with adequate the RC shield. This rectum volume was delineated to be contracted away from the target by 6-8 mm and 8-10 mm for patients in supine and prone positioning respectively. Since this volume is defined only for planning purpose, with no role in planning evaluation, we named it as “visual rectum volume” (VRV).

**B. Dose prescription**

For SIMAT plans, the prescribed dose in phases I, II & III were 54 Gy in 27 fractions, 12 Gy in 6 fractions and 12 Gy in 6 fractions, respectively [20]. For M-DAT plans, the prescribed doses for the PPO and PSV were 78 Gy and 61.23 Gy in 39 fractions. The prescribed dose to the PSV is equivalent to 54 Gy in 27 fractions as calculated using a computerized version of Orton's time-dose-fractionation (TDF) tables [31] (contributed by Maria A. Czerminska, University of Illinois, Radiology, Chicago).

**C. SIMAT technique**

Three phase SIMAT plans had been performed and calculated using CT images of the 10 supine patients. In the first phase (phase I) the bilateral arcs covered the PSV however it covered the PPO in the other two phases. In phases I & II non-shielded arcs of ranges 45° to 145° for left arcs and 215° to 315° for the right arcs were used. In phase III shielded RC arcs of ranges 90° to 180° for left arcs and 180° to 270° for the right arcs were used. A margin of 5 mm between field edges and the PTV in all directions was taken [20]. The SIMAT plan was produced by the sum of the three phases using the “plan sum” option of Eclipse planning system.

**D. M-DAT Technique**

Two arcs covering 350° (one clockwise and the other anti-clockwise) were generated automatically by the planning system to fit the PTV and shield the VRV. The two conformal fields of symmetrical angles with respect to laterals were assigned to cover the PPO. The target volume dose uniformity is produced by the combination of a high dose volume yielded anteriorly by the arcs with that yielded posteriorly by the conformal fields as shown in [Fig.1.](../fig1.doc) The margins of the MLC aperture to the PTVs for all fields were taken 5 mm in all directions except for in the superior of the PSV (for arcs) and the inferior of the PPO (for arcs and static fields) it was 8 mm. This was adequate to cover 100 % of the PTVs with 95% of the prescribed doses. Three gantry angles 0^o^, 15^o^ and 30^o^ (A0, A15 and A30) downwards to laterals for supine patients and upwards for prone were employed in a trial and error fashion to find the optimum conformal field angles. It was thought that applying wedges (anteriorly oriented thick ends) to the two conformal fields with lowering of their weighting might help in sparing of femoral heads. For optimization purposes a set of enhanced dynamic wedges of angles 0°, 30°, 45° and 60° (W0 to W60) were tested in a trial and error fashion to find the optimum gantry angle. The dose to the PSV was adjusted, in some cases, by the use of a hard wedge of angle 30° with shielded arcs such that its thin part was oriented superiorly (in the PSV direction).

**E. Plan evaluation and comparisons**

The dose volume histograms (DVHs) for the PTVs and organs at risk were used for plan evaluation and comparisons. All plans were normalized based on the DVHs to ensure that 95% of the PTV received 100 % of the prescribed dose. The doses inhomogeneity (DI) in the PTV was defined as (D_5_-D_95_)/D_mean_ [32], D_5_, D_95_ and D_mean_ are defined in Table 1.

The M-DAT plans were considered acceptable if they satisfied the dose guidelines designed by the Radiation Therapy Oncology Group (RTOG – 0126) in patients treated for localized prostate cancer [33]. According to these guidelines, rectal criteria require that no more than 15%, 25%, 35% and 50% of the rectum volume should receive more than 75 Gy, 70 Gy, 65 Gy and 60 Gy, respectively. For the bladder, it is required that no more than 15%, 25%, 35% and 50% of the bladder volume should receive more than 80 Gy, 75 Gy, 70 Gy and 65 Gy, respectively. In addition, no more than 2 % of the PTV is to exceed 84.7 Gy and not less than 98 % covered by the prescribed dose (78 Gy for PPO and 54 Gy for PSV). The femoral head doses are not mentioned in RTOG-0126. We applied the recommended criteria for the accepted femoral heads doses by ICRU Report 62 [34] in which the volume that covered by 52 Gy or more should be minimized.

The definitions of doses and volumes used for optimization and comparison are listed in [table](../Table1.doc) (1). The mean DVHs were taken for supine and prone patients separately. M-DAT was applied for supine and prone patients while SIMAT was calculated for supine patients only, and then results were compared.

**F. Plan verification**

Ion chamber and film dosimetry were used to verify the calculated dose by the Eclipse planning system for the M-DAT plans. The plans for all patients were exported in Eclipse to the CT images of cylindrical phantom type PTW- T9193 (PTW Freiburg, Germany) and calculated for comparison with ion chamber measurements. The plans of all supine and prone patients were exported also to the CT images of Alderson Rando anthropomorphic phantom (from PTW Freiburg, Germany) (Alderson phantom) and calculated for comparison with film measurements.

Pinpoint ion chamber 0.015 cc type PTW- 31006 located in the central axis of the cylindrical phantom was used to verify the calculated isocenter dose. The measurements were performed at the levels that corresponded to the central axial levels of prostate and seminal vesicles in the phantom and then compared with the calculated doses at these points.

Before film calibration, the machine output of the 6MV photon beam was adjusted such that the beam output corresponds to 1 cGy/MU at d_max_ for a 10 × 10 cm^2^ field at SSD = 100 cm. A calibration set of five Kodak Extended Dose Range (EDR2) was inserted individually in the spacing of the central slabs of a solid water phantom (30 x 30 x 30 cm^3^) type PTW-29672. The phantom was positioned to locate the film in the beam central axis plane at 90^o^ gantry angle with the field size 10 x 10 cm^2^ and SSD = 100 cm. The films were exposed to the 6MV photon beam with five monitor unit values (50, 200, 400, 700 and 1000 MU) then processed 1 h after irradiation [35] by automatic processing machine (AFP Imaging CORP, USA) and scanned by Vidar-16 scanner (Vidar Systems Corporation, Herndon, VA). The relation between the depth and the optical density (OD) for each calibration film were performed by PTW-Film Analysis 1.3 software and exported to a Microsoft Excel file.

To get the relationship between the dose and OD, the percentage depth dose data of the 6MV photon beam for the field size 10 x 10 cm^2^ at SSD = 100 cm was converted into five relations between depth and dose each one corresponds to one of the used monitor unit values using the same Excel file. So that, at all depths in each film, one can determine how OD corresponds to dose. To reduce the variability of working conditions, the calibration and dosimetry measurements were performed in a single session [36].

For the M-DAT plan verification, two films were inserted axially in the Alderson phantom in two levels located in the regions of prostate and seminal vesicles of each patient of interest. The measured dose distributions by films and that calculated by Eclipse at the same levels were imported and compared using PTW-Varisoft 3.1. The criterion employed to evaluate the accuracy of Eclipse calculations was the gamma index [37], with individual acceptance criteria of 3 % dose difference (DD) and 4 mm distance to agreement (DTA) (3% DD and 4mm DTA). Scanned and calculated area of 10 x 10 cm2 was found to be adequate for including the isodose lines higher that 50%. A quantitative analysis of the dose distribution comparison based on gamma reports (a report contains the total No. of evaluated pixels and percentage passed and failed pixels) was performed to show the percentage pixels in the distribution that exceed the acceptance criteria (the percentage failed pixels) for both prostate and seminal vesicles level for supine and prone patients.

**III. RESULTS AND DISCUSSION**

**A. SIMAT plan**

Our results of SIMAT planning are in agreement with that previously published [20]. With respect to the PPO coverage, the calculated D_max_ and D_mean_ indicate that the results of this study are higher by only 2.44 % and 2.28 %. Also, the values of D_max_ in case of the RC and LF here are lower only by 1.47 % and 3.62 %, respectively. Our results indicated that SIMAT plan could be more protective to the RC and LF than that previously published [20] as D_mean_ are lower by 7.10 % and 17.36 %, respectively. The large difference of D_mean_ for the LF could be due to its definition since it is delineated in this study as the head and neck of femur while in the previous publication, [21], it was delineated as the head only.

**B. M-DAT Plan optimization**

Regarding M-DAT plan optimization, examples of DVHs of all volumes of interest with three conformal field angles A0, A15 and A30 and different dynamic wedge angles W30, W45 and W60 are presented in figures [2](../fig%202C.doc) and [3](../fig%203.doc). Because of the symmetrical field arrangement of the M-DAT plan, the DVHs of the femoral heads are represented only by the LF. The effect of variation of the conformal field angles on the DVHs of the PPO, RC and LF are demonstrated in [Fig. 2](../fig%202C.doc). The figures showed that as the angle increases, the RC dose increases, the LF dose decreases with minimal change in the PPO coverage. Reasonably, as the two conformal fields moved posteriorly (downwards for supine patients or upwards for prone), their crossing volume in rectum increases ([Fig. 2a](../fig%202C.doc)) and their coverage of the femoral heads decrease ([Fig. 2](../fig%202C.doc)b). Consequently, the angle A0 is optimum with respect to rectum protection while, it is worse with respect to femoral head protection. This is due to the undesirable alignment of the two conformal fields with the two femoral heads at this angle. Therefore, A15 is chosen to be the optimum gantry angle for the conformal fields.

To reduce the LF doses, enhanced dynamic wedges were used such that their thick ends were oriented anteriorly in both conformal fields. This enabled us to reduce the weighting of conformal fields which significantly reduce the LF dose with A15 gantry angle as shown in Fig.2b. Further reduction of the dose to the LF was obtained with greater wedge angles with more reduction of the weighting of the conformal fields as shown in [Fig. 2b](../fig%202C.doc). On the other hand, the doses to the PSV and BL increased as the wedge angle increased with the same gantry angle as shown in [Fig. 3](../fig%203.doc). This is due to the enhancement of the dose contribution of the shielded arcs as a result of the reduction of the weighting of the conformal fields. Such enhancement was not sufficient to cover the seminal vesicles with the prescribed dose for obese patients particularly in supine positioning. This is due to the extra attenuation carried out by the abdominal fat strip that faced the PSV with shielded arcs. Consequently, the 30° hard wedge of thin end oriented to the PSV was used with shielded arcs to increase the dose to the PSV volume on the expense of the dose to the PPO volume. The plan was then renormalized to achieve the desired dose coverage to both the PPO and PSV volumes (100 % of the PTVs covered by 95 % of the prescribed dose). Typical parameters of optimized M-DAT are presented in [table](../Table2.doc) (2).

**C. Plan comparisons**

The comparison of the dose distribution to the PPO and PSV indicated that SIMAT and M-DAT for supine patients are similar except for the PO dose homogeneity (represented by the DI) which was better in SIMAT as shown in table 3. It illustrates also that the PSV dose homogeneity in SIMAT was better than in M-DAT while the dose coverage (represented by the mean, modal and median) in M-DAT was better.

No significant variations are noted between M-DAT for supine and prone patients with respect to the PPO and PSV dose distribution. The values of D_max_ in [Table 3](../Table%2034.doc) indicated that no volume of the PPO is covered by 84.7 Gy (D_max_ < 84.7). This is in agreement with the RTOG-0126 criteria for the maximum dose coverage to the target. The rectum protection in SIMAT is worse compared with M-DAT in supine positioning, while M-DAT in prone positioning is the best as shown in [Fig 4](../Fig4&5.doc)a. This is due the enlargement of the rectum size posteriorly in prone positioning which resulted in displacement of most of the rectal wall away from the treatment volume as noted in comparisons of the rectum shape in CT images of all supine and prone patients under study.

[Table 3](../Table%2034.doc) indicates that all the statistical parameters of rectum dose distribution for prone patients are superior with respect to rectum protection except for the D_max_ as shown also in [Fig](../fig%202.doc). 2. This is due to the extra margin (by 3 mm) taken in all directions to the PO and SV in the construction of the PPO and PSV to consider the potential for breathing to influence prostate movement for patients in prone positioning.

On the other hand SIMAT is better than M-DAT in both supine and prone positioning with respect to low doses (below 55 Gy) protection to the bladder as shown in [Fig](../Fig4&5.doc) 4b. This is a consequence of the higher delivered doses to the PSV volume in M-DAT (table 3). [Fig](../Fig4&5.doc) 4b also shows that the bladder doses are not influenced by the patient positioning with M-DAT technique.

[Table 4](../Table%2034.doc) demonstrates the quantitative analysis of the DVHs based on our comparison criteria mentioned above and tabulated in [table](../Table1.doc) (1). Accordingly, the doses to 15%, 25%, 35% and 50% of the rectum volume with M-DAT in supine positioning are 7.7 %, 18.2 %, 22.4 % and 28.5 % lower than that of SIMAT, while they are higher than that of M-DAT in prone positioning by 17.3 %, 13.8 %, 12.4 % and 13.5% of the prescribed dose, respectively.

e

The percentage rectum volumes covered by 50 and 60 Gy with M-DAT in supine positioning are 18 % and 9 % lower than that of SIMAT, while they are higher than that of M-DAT in prone positioning by 10.0 % and 6 %, respectively.

Tables 3 and 4 indicate that the LF volume that is covered by doses exceed 52 Gy in both SIMAT and M-DAT techniques are negligible. Consequently, they are accepted with respect to LF protection according to ICRU Report 62 criteria.

Follow up of all patients showed clinical regression of prostatic tumor as evidenced by CT imaging and 3 month interval measurement of their PSA level in their blood. All patients were weaned off total androgen blockade in a period of 6 – 12 months following radical radiotherapy. Acute cystitis during treatment was tolerable and was treated symptomatically. Rectal and bladder complication were minimal after one year follow up. According to our results and the pervious studies [38-40], the predictive late rectal bleeding after M-DAT in prone positioning is reasonably lower than both SIMAT and M-DAT in supine positioning. These studies had shown that late rectal toxicity is significantly correlated with the absolute/percentage volume of the rectum receiving all ranges of dose.

**D. Plan Verification**

The absolute dose measurements performed at the isocentric axis of the cylindrical phantom indicated that the dose values at the chosen levels that represent prostate and seminal vesicles are in agreement with the calculated doses at the same points. Fig. 5 shows the percentage variation of the calculated and measured doses in the levels of prostate and seminal vesicles for ten supine (1 to 10) and prone (11 to 20) patients including four cases (3, 4, 6 and 17) of wedged arcs. In the prostate level the mean variation between the measured and calculated doses and maximum discrepancy are 1.7% and 3%, respectively. However, in the level of seminal vesicles they are 2.6% and -3.5%, respectively. The maximum discrepancies are noted when the wedged arcs are applied.

Fig. 6 shows an example of gamma distributions and the measured and calculated dose distributions for prostate and seminal vesicles levels. It is noted that the main region of the dose distribution that failed the acceptance criteria (3% DD and 4mm DTA) can be observed in the low-dose regions, particularly in level of seminal vesicles. Fig. 7 shows the percentage failed pixels in the distribution for both prostate and seminal vesicles levels for the supine (patient's No. 1-10) and prone patients (patient's No. 11-20). It is easy to note that at the prostate level the dose distribution is more accurately calculated than at the seminal vesicles level. In all cases, less than 10 % of the pixels failed the acceptance criteria even with wedged arcs (patient's No. 3, 4, 6 and 17).

**IV. CONCLUSION**

The optimized single phase M-DAT technique produced distinctly favorable dose distributions for the prostate and seminal vesicles coverage as well as rectum and femoral heads protection. In addition, the desired concave dose distribution is obtained by a single phase arc therapy technique based on forward planning dose calculation. Compared with SIMAT treatments, the M-DAT treatments produced less bladder protection in the region of lower doses with consistently greater sparing of the rectum in regions of higher and lower doses. When the treatment of the prostate only is required, the present technique can be applied by removing the seminal vesicles from the dynamic arcs apertures. Generally, the M-DAT technique carries the advantages of arc therapy in the treatment of central lesion as well as the maximum protection for long organ at risk located near to the lesion of interest. This technique may be investigated for spinal cord protection in the treatment of esophageal, paraortic and head and neck lesions.

**ACKNOWLEDGEMENT**

To our consultants and colleges in the departments of radiation physics and radiotherapy in Maadi Armed Forces Medical Compound especially the Prof. Dr. Mossad Hegazy for his radiobiological background supports.

**REFERENCES**

1. Hanks G, Marta K and Diamond J. The effect of dose on local control of prostate cancer. Int J Radiat Oncol Biol Phys 1988; 15:1299–1305.
2. [Smit W](http://www.ncbi.nlm.nih.gov/sites/entrez?Db=pubmed&Cmd=Search&Term="Smit WG"%5BAuthor%5D&itool=EntrezSystem2.PEntrez.Pubmed.Pubmed_ResultsPanel.Pubmed_RVAbstractPlus), [Helle P](http://www.ncbi.nlm.nih.gov/sites/entrez?Db=pubmed&Cmd=Search&Term="Helle PA"%5BAuthor%5D&itool=EntrezSystem2.PEntrez.Pubmed.Pubmed_ResultsPanel.Pubmed_RVAbstractPlus), [van Putten W](http://www.ncbi.nlm.nih.gov/sites/entrez?Db=pubmed&Cmd=Search&Term="van Putten WL"%5BAuthor%5D&itool=EntrezSystem2.PEntrez.Pubmed.Pubmed_ResultsPanel.Pubmed_RVAbstractPlus), [Wijnmaalen A](http://www.ncbi.nlm.nih.gov/sites/entrez?Db=pubmed&Cmd=Search&Term="Wijnmaalen AJ"%5BAuthor%5D&itool=EntrezSystem2.PEntrez.Pubmed.Pubmed_ResultsPanel.Pubmed_RVAbstractPlus), [Seldenrath J](http://www.ncbi.nlm.nih.gov/sites/entrez?Db=pubmed&Cmd=Search&Term="Seldenrath JJ"%5BAuthor%5D&itool=EntrezSystem2.PEntrez.Pubmed.Pubmed_ResultsPanel.Pubmed_RVAbstractPlus) and [van der Werf-Messing B](http://www.ncbi.nlm.nih.gov/sites/entrez?Db=pubmed&Cmd=Search&Term="van der Werf-Messing BH"%5BAuthor%5D&itool=EntrezSystem2.PEntrez.Pubmed.Pubmed_ResultsPanel.Pubmed_RVAbstractPlus). Late radiation damage in prostate cancer patients treated by high dose external radiotherapy in relation to rectal dose. Int J Radiat Oncol Biol Phys. 1990; 18: 23–29.
3. Zelefsky M, Leibel S, Gaudin P, Kutcher G, Fleshner N, Venkatramen E, Reuter V, Fair W, Ling C and Fuks Z. Dose escalation with three-dimensional conformal radiation therapy affects the outcome in prostate cancer. [Int J Radiat Oncol Biol Phys](http://www.sciencedirect.com/science?_ob=JournalURL&_cdi=5070&_auth=y&_acct=C000050221&_version=1&_urlVersion=0&_userid=10&md5=f599655d799c1a72f1644ec1617b7cf3). 1998;41: 491-500
4. Perez C, Michalski J, Mansur D and Lockett M. Three-dimensional conformal therapy versus standard radiation therapy in localized carcinoma of prostate: an update. Clinical Prostate Cancer 2002; 1:97-104.
5. Perez C, Purdy J, Harms W and Gerber R. Three-dimensional treatment planning and conformal radiation therapy. Radiother. Oncol. 1995; 1:32-36.
6. Brundage M, Lukka H, Crook J, Warde P, Bauman G, Catton C, Markman B, Charette M and Cancer Care Ontario Practice Guidelines Initiative Genitourinary Cancer Disease Site Group. The use of conformal radiotherapy and the selection of radiation dose in T1 or T2 low or intermediate risk prostate cancer – a systematic review.  [Radiother. Oncol](http://www.sciencedirect.com/science?_ob=JournalURL&_cdi=5155&_auth=y&_acct=C000050221&_version=1&_urlVersion=0&_userid=10&md5=249defed4c7340a36ac35076b5590f4f)  2002; 64:239-250.
7. Chism D, Horwitz E, Hanlon A, Pinover W, Mitra R, and Hanks G. Late morbidity profiles in prostate cancer patients treated to 79–84 Gy by a simple four-field coplanar beam arrangement. [Int J Radiat Oncol Biol Phys](http://www.sciencedirect.com/science?_ob=JournalURL&_cdi=5070&_auth=y&_acct=C000050221&_version=1&_urlVersion=0&_userid=10&md5=f599655d799c1a72f1644ec1617b7cf3). 2003; 55:71-77.
8. Horine P, Roach M, Pickett B, Phillips T and Verhey L. Optimization of the oblique angles in the treatment of prostate cancer during six-field conformal. Medical Dosimetry. 1994; 19:237-254.
9. Luka S and Kurup R. Comparison of treatment plans for irradiating adenocarcinoma of the prostate. Medical Dosimetry. 1995; 20:117-122.
10. Fiorino C, Reni M, Cattaneo G, Bolognesi A and Calandrino R. Comparing 3-, 4- and 6-fields techniques for conformal irradiation of prostate and seminal vesicles using dose-volume histograms. [Radiother Oncol.](http://www.sciencedirect.com/science?_ob=JournalURL&_cdi=5155&_auth=y&_acct=C000050221&_version=1&_urlVersion=0&_userid=10&md5=249defed4c7340a36ac35076b5590f4f)  1997; 44: 251-257.
11. Serago C, Lewin A, Houdek V, Schwade G and Abitbol A. Multiplanar arc boost radiation therapy for prostate cancer. Radiology. 1989; 172(2): 561-564.
12. Bedford J, Khoo V, Oldham M, Dearnaley D and Webb S. A comparison of coplanar four-field techniques for conformal radiotherapy of the prostate. [Radiother. Oncol.](http://www.sciencedirect.com/science?_ob=JournalURL&_cdi=5155&_auth=y&_acct=C000050221&_version=1&_urlVersion=0&_userid=10&md5=249defed4c7340a36ac35076b5590f4f)  1999;[5](http://www.sciencedirect.com/science?_ob=IssueURL&_tockey=%23TOC%235155%231999%23999489996%23106863%23FLA%23display%23Volume_51,_Issue_3,_Pages_197-292_(1_June_1999)%23tagged%23Volume%23first%3D51%23Issue%23first%3D3%23Pages%23first%3D197%23last%3D292%23date%23(1_June_1999)%23&_auth=y&view=c&_acct=C000050221&_version=1&_urlVersion=0&_userid=10&md5=ea913f26e82496b0214cf73db8968878):225-235.
13. Bedford J, Khoo V, Webb S and Dearnaley D. Optimization of coplanar six-field techniques for conformal radiotherapy of the prostate. [Int J Radiat Oncol Biol Phys](http://www.sciencedirect.com/science?_ob=JournalURL&_cdi=5070&_auth=y&_acct=C000050221&_version=1&_urlVersion=0&_userid=10&md5=f599655d799c1a72f1644ec1617b7cf3). 2000; 46:231-238.
14. Scandolaro L, Bossi A, Ostinelli A, Marinoni M and Isella E. Exclusive irradiation of clinically localized prostatic carcinoma: comparison with various techniques. Radiologia Medica. 1997;82-89.
15. William H, Daniel C, Gregory G, John L, Michael K, Christine B and Anatoly D. Limitations of reduced-field irradiated volume and technique in conventional radiation therapy of prostate cancer: Implications for conformal 3-D treatment. International Journal of Cancer 2000; 90 265-274.
16. Akazawa P, Roach M, Pickett B, Purser P, Parkinson D, Rathbun C and Margolis L. Three dimensional comparison of blocked arcs vs. four and six field conformal treatment of the prostate. Radiotherapy and Oncology, 1996; 41:83-88.
17. Roach M, Akazawa P, Pickett B, Purser P, Parkinson D, Meyler T and Margolis L. Bilateral arcs using "averaged beam's eye views": a simplified technique for delivering 3-D based conformal radiotherapy. Medical dosimetery. 1994; 19:159-168.
18. Weil M, Crawford E, Cornish P, Dzingle W, Stuhr K, Pickett B and Roach M. Minimal toxicity with 3-FAT radiotherapy of prostate cancer. Seminars In Urologic Oncology , 2000;18:127-132
19. Wong E, Chen J and Greenland J. Intensity-modulated arc therapy simplified [Int J Radiat Oncol Biol Phys](http://www.sciencedirect.com/science?_ob=JournalURL&_cdi=5070&_auth=y&_acct=C000050221&_version=1&_urlVersion=0&_userid=10&md5=f599655d799c1a72f1644ec1617b7cf3). 2002; 53:222-235.
20. Bauman G, Gete E, Chen J and Wong E. Simplified intensity-modulated arc therapy for dose escalated prostate cancer radiotherapy. Medical dosimetry, 2004; 29:18-25.
21. Fiorino[a](http://www.sciencedirect.com/science?_ob=ArticleURL&_udi=B6TBY-3VS7F16-4&_user=10&_coverDate=08%2F31%2F1998&_fmt=full&_orig=browse&_cdi=5155&view=c&_acct=C000050221&_version=1&_urlVersion=0&_userid=10&md5=2a35b23565b6b16f4050660136097019&ref=full#aff1) C, Reni[b](http://www.sciencedirect.com/science?_ob=ArticleURL&_udi=B6TBY-3VS7F16-4&_user=10&_coverDate=08%2F31%2F1998&_fmt=full&_orig=browse&_cdi=5155&view=c&_acct=C000050221&_version=1&_urlVersion=0&_userid=10&md5=2a35b23565b6b16f4050660136097019&ref=full#aff2) M, Bolognesi A, Bonini A, Cattaneo G and Calandrino R. Set-up error in supine-positioned patients immobilized with two different modalities during conformal radiotherapy of prostate cancer.  [Radiotherapy and Oncology, 1998; 49: 133-141.](http://www.sciencedirect.com/science?_ob=JournalURL&_cdi=5155&_auth=y&_acct=C000050221&_version=1&_urlVersion=0&_userid=10&md5=249defed4c7340a36ac35076b5590f4f)
22. Tinger A, Michalski J, Cheng A, Low D, Zhu R, Bosch W, Purdy J and Perez C. A critical evaluation of the planning target volume for 3-D conformal radiotherapy of prostate cancer. Int J Radiat Oncol Biol Phys. 1998; 42: 213-221.
23. Antolak J, Rosen I, Childress C, Zagars G and Pollack A. Prostate target volume variations during a course of radiotherapy. Int J Radiat Oncol Biol Phys. 1998; 42: 661-672.
24. Dawson L, Mah K, Franssen E and Morton G. Target position variability throughout prostate radiotherapy. Int J Radiat Oncol Biol Phys. 1998; 42: 1155-1161.
25. Zelefsky M, Crean D, Mageras G, Lyass O, Happersett L, Ling C, Leibel S, Fuks Z, Bull S, Kooy H, Herk M and Kutcher G. Quantification and predictors of prostate position variability in 50 patients evaluated with multiple CT scans during conformal radiotherapy.  [Radiotherapy and Oncology, 1999; 50: 225-234.](http://www.sciencedirect.com/science?_ob=JournalURL&_cdi=5155&_auth=y&_acct=C000050221&_version=1&_urlVersion=0&_userid=10&md5=249defed4c7340a36ac35076b5590f4f)
26. [Stroom J](http://www4.infotrieve.com/newmedline/summary.asp?presearch=1&term=Stroom JC) , [Koper P](http://www4.infotrieve.com/newmedline/summary.asp?presearch=1&term=Koper PC), [Korevaar G,](http://www4.infotrieve.com/newmedline/summary.asp?presearch=1&term=Korevaar GA) [van Os M](http://www4.infotrieve.com/newmedline/summary.asp?presearch=1&term=van Os M) , [Janssen M](http://www4.infotrieve.com/newmedline/summary.asp?presearch=1&term=Janssen M) , [de Boer H,](http://www4.infotrieve.com/newmedline/summary.asp?presearch=1&term=de Boer HC) [Levendag P](http://www4.infotrieve.com/newmedline/summary.asp?presearch=1&term=Levendag PC) and Heijmen B. Internal organ motion in prostate cancer patients treated in prone and supine treatment position. Radiother Oncol, 1999; 51: 237-48.
27. [Miralbell R](http://www4.infotrieve.com/newmedline/summary.asp?presearch=1&term=Miralbell R), [Ozsoy O](http://www4.infotrieve.com/newmedline/summary.asp?presearch=1&term=Ozsoy O), [Pugliesi A](http://www4.infotrieve.com/newmedline/summary.asp?presearch=1&term=Pugliesi A), [Carballo N](http://www4.infotrieve.com/newmedline/summary.asp?presearch=1&term=Carballo N), [Arnalte R](http://www4.infotrieve.com/newmedline/summary.asp?presearch=1&term=Arnalte R), [Escud L](http://www4.infotrieve.com/newmedline/summary.asp?presearch=1&term=EscudÃ© L), [Jargy C](http://www4.infotrieve.com/newmedline/summary.asp?presearch=1&term=Jargy C), [Nouet P](http://www4.infotrieve.com/newmedline/summary.asp?presearch=1&term=Nouet P) and [Rouzaud M](http://www4.infotrieve.com/newmedline/summary.asp?presearch=1&term=Rouzaud M). Dosimetric implications of changes in patient repositioning and organ motion in conformal radiotherapy for prostate cancer. Radiother Oncol, 2003; 66: 197-202.
28. [Little D](http://www4.infotrieve.com/newmedline/summary.asp?presearch=1&term=Little DJ), [Dong L](http://www4.infotrieve.com/newmedline/summary.asp?presearch=1&term=Dong L), [Levy L](http://www4.infotrieve.com/newmedline/summary.asp?presearch=1&term=Levy LB), [Chandra A](http://www4.infotrieve.com/newmedline/summary.asp?presearch=1&term=Chandra A) and Kuban D. Use of portal images and BAT ultrasonography to measure setup error and organ motion for prostate IMRT: implications for treatment margins. Int J Radiat Oncol Biol Phys. 2003; 56: 1218-1224.
29. [Jackson A](http://www4.infotrieve.com/newmedline/summary.asp?presearch=1&term=Jackson A) , Partial irradiation of the rectum. Semin Radiat Oncol, 2001;11(3): 215-23.
30. [Dawson L](http://www.ncbi.nlm.nih.gov/entrez/query.fcgi?db=pubmed&cmd=Search&itool=pubmed_Abstract&term="Dawson+LA"%5BAuthor%5D), [Litzenberg D](http://www.ncbi.nlm.nih.gov/entrez/query.fcgi?db=pubmed&cmd=Search&itool=pubmed_Abstract&term="Litzenberg+DW"%5BAuthor%5D), Brock K, Sanda M, Sullivan M, Sandler H and Balter J. A comparison of ventilatory prostate movement in four treatment positions. [Int J Radiat Oncol Biol Phys.](javascript:AL_get(this, 'jour', 'Int J Radiat Oncol Biol Phys.');) 2000; 2: 319-23.
31. Orton C and Ellis F. A simplification in the use of NSD concept in practical radiotherapy. British journal of radiology, 1973; 46: 529-537.
32. Mock U, Georg D, Bogner J, Auberger T and Pötter R. Treatment planning comparison of conventional, 3D conformal and intensity modulated photon (IMRT) and proton therapy for paranasal sinus carcinoma. Int J Radiat Oncol Biol Phys. 2004; 58: 147-154.
33. Michalski J, Purdy J, Bruner D and Amin M. A phase III randomized of high dose 3D-CRT/IMRT versus Standard dose 3D-CRT/IMRT in Patients treated for localized prostate cancer. Radiation Therapy Oncology Group (RTOG-0126) 2004.
34. Wambersie A and Landberg T. Prescribing, recording and reporting photon beam therapy. Supplement to ICRU Report 50, ICRU Report 62(1999).
35. [Childress](http://www.ncbi.nlm.nih.gov/sites/entrez?Db=pubmed&Cmd=Search&Term="Childress NL"%5BAuthor%5D&itool=EntrezSystem2.PEntrez.Pubmed.Pubmed_ResultsPanel.Pubmed_RVAbstractPlus) N and [Rosen I](http://www.ncbi.nlm.nih.gov/sites/entrez?Db=pubmed&Cmd=Search&Term="Rosen II"%5BAuthor%5D&itool=EntrezSystem2.PEntrez.Pubmed.Pubmed_ResultsPanel.Pubmed_RVAbstractPlus). Effect of processing time delay on the dose response of Kodak EDR2 film. Med Phys. 2004; 31(8):2284-2288.
36. Yeo I, Wang C, et al. A filtration method for improving film dosimetry in photon radiation therapy. Med Phys. 1997; 24(12):1943-1953.
37. Low D, Harms W, Mutic S and Purdy J. A technique for the quantitative evaluation of dose distributions. Med Phys. 1996; 25(5):656-661.
38. Jackson A, Skwarchuk M, Zelefsky M, Cowen D, Venkatraman E, Levegrun S, Burman C, Kutcher G, Fuks Z, Liebel S and Ling C. Late rectal bleeding after conformal radiotherapy of prostate cancer (II): volume effects and dose - volume histograms Int J Radiat Oncol Biol Phys. 2001; 49: 685-698.
39. Wachter S, Geratner N, Goldner G, Pötzi R, Wambersie A and Pötter R. Rectal Sequelae after conformal radiotherapy of prostate cancer: dose-volume histograms as predictive factors. Radiother Oncol, 2001; 59: 65-70.
40. Fiorino C, Cazzarini C, Vavassori V, Sanguineti G, Bianchi C, Cattaneo G, Foppiano F, Magli A and Piazzolla A. Relationships between DVHs and late rectal bleeding after radiotherapy of prostate cancer: analysis of a large group of patients pooled from three institutes. Radiother Oncol, 2002; 64: 1-12.

Figure Legends

Fig. 1. Fields arrangement and isodose distributions of (a) the two shielded arcs, (b) bilateral conformal beams and their (c) combination.

Fig. 2. DVHs of a supine patient for (a) PPO and RC with gantry angles A0, A15 and A30 and RC with gantry angle A15 when different dynamic wedges (W30, W45 and W60) were used and (b) LF with gantry angles A0, A15 and A30 and PPO and LF with gantry angle A15 when different dynamic wedges (W30, W45 and W60) were used.

Fig. 3. DVHs of a supine patient for PSV and BL with gantry angle A15 when different dynamic wedges (W30, W45 and W60) were used.

Figure 4: Plot of mean DVHs of 10 supine and 10 prone patients for (a) rectum and (b) bladder with SIMAT and M-DAT.

Fig. 5. The percentage variation of the calculated and measured doses in the levels of prostate and seminal vesicles for the ten supine (1 to 10) and prone (11 to 20) patients including four cases (3, 4, 6 and 17) of wedged arcs

Fig. 6. An example of gamma distribution (upper parts) and the measured and calculated M-DAT dose distributions (lower parts) for prostate (left side) and seminal vesicles (right side) levels for a 10 x 10 cm^2^ scanned and calculated area (the coordinate axes are in a scale of 10 mm). The green areas indicate regions where pixels passed the gamma acceptance criteria (3% DD and 3 mm DTA) while the red indicate regions where pixels failed. The continuous and dashed lines represent the measured and calculated dose distributions, respectively.

Fig. 7. Percentage of failed pixels in M-DAT dose distribution compared to the gamma acceptance criteria (3% DD and 4 mm DTA) for prostate and seminal vesicles levels of ten supine (1 to 10) and prone (11 to 20) patients including four cases (3, 4, 6 and 17) of wedged arcs.

| Table 2 Optimum setup parameters of M-DAT plan for Varian 23 EX machine [in the Varian IEC scale]) | | | | | |
| --- | --- | --- | --- | --- | --- |
|  | VRV  shielding | Gantry | Collimator | Wedge | Weighting |
| ARC1 | Yes | 175 – 185 Anti-clock wise | 0 | Hard wedge 30 IN (in four cases) | 1.5 to 1.65 |
| ARC2 | Yes | 185 – 175 Clock wise | 0 | Hard wedge 30 IN (in four cases) | 1.5 to 1.65 |
| LT | No | 105 | 90 | EDW 45 or 60 IN | 0.40 to 0.55 |
| RT | No | 255 | 90 | EDW 45 or 60 OUT | 0.40 to 0.55 |
| The normalization condition was 100 % of the PTVs covered by 95 % of the prescribed dose | | | | | |

| Table (1) Dose and volume comparison metrics. | |
| --- | --- |
|  | Definition |
| D_max_ ,D_min_ ,D_mean_ , D_modal_ and D_median_  V_84_  V_Dmin_  D_5_, D_15_, D_25_, D_35_ D_50_ and D_95_  V_52_, V_50_ and V_60_ | The maximum, minimum, mean, modal and median doses to volume of interest.  The percentage PTV covered by 84 Gy.  The percentage volumes of minimum doses.  The doses to5%, 15%, 25%, 35%, 50 % and 95% of the volume of interest.  The parentage of any volume covered by 52, 50 and 60 Gy. |

| Table 3. Dose comparison of M-DAT and SIMAT plans concerning all regions of interest for 10 supine and 10 prone patients. | | | | | | | | | | | | |
| --- | --- | --- | --- | --- | --- | --- | --- | --- | --- | --- | --- | --- |
|  | PPO | | PSV | | RC | | | BL | | | LF | |
|  | SIMAT  mean  (± SD) | M-DAT  Supine and Prone  mean  (± SD) | SIMAT  mean  (± SD) | M-DAT  Supine and Prone  mean  (± SD) | SIMAT  mean  (± SD) | M-DAT | | SIMAT  mean  (± SD) | M-DAT | | SIMAT  mean  (± SD) | M-DAT  Supine and Prone  mean  (± SD) |
|  |  |  |  |  |  | Supine  mean  (± SD) | Prone  mean  (± SD) |  | Supine  mean  (± SD) | Prone  mean  (± SD) |  |  |
| D_min_ (Gy) | 73.92  (± 5.2) | 73.92  (±1.0) | 50.0  (± 3.2) | 52.9  (± 2.5) | 4.19  (± 1.4) | 3.77  (± 1.4) | 1.4  (± 0.5) | 2.15  (± 1.2) | 8.91  (± 5.0) | 6.5  (± 4.0) | 4.39  (± 4.1) | 3.76  (± 2.0) |
| D_max_ (Gy) | 81.00  (± 0.8) | 83.10  (± 0.2) | 78.04  (± 1.2) | 80.05  (± 1.5) | 70.65  (± 8.2) | 70.51  (± 7.3) | 78.8  (± 3.4) | 76.37  (± 0.9) | 79.37  (± 2.6) | 78.8  (± 3.2) | 57.48  (± 14.2) | 56.80  (± 4.2) |
| D_mean_ (Gy) | 77.88  (± 0.2) | 77.88  (± 0.4) | 54.79  (± 1.6) | 58.9  (± 3.3) | 39.36  (± 6.0) | 30.22  (± 5.1) | 22.5  (± 5.1) | 25.85  (± 5.1) | 34.05  (± 8.5) | 32.8  (± 8.0) | 27.16  (± 5.6) | 27.41  (± 6.1) |
| D_median_ (Gy) | 78.00  (± 0.2) | 78.00  (± 0.4) | 52.01  (± 0.7) | 56.5  (± 3.7) | 40.84  (± 8.1) | 26.99  (± 5.8) | 16.9  (±6.0) | 20.36  (± 12.4) | 31.52  (± 9.4) | 28.2  (± 8.1) | 26.28  (±11.4) | 27.33  (± 8.4) |
| D_modal_ (Gy) | 78.42  (± 1.3) | 78.42  (± 1.3) | 52.05  (± 0.4) | 57.48  (± 1.0) | 54.73  (± 10.2) | 20.94  (± 7.6) | 18.9  (± 4.0) | 13.53  (± 5.6) | 23.62  (± 8.8) | 20.7  (±7.2) | 24.97  (± 5.6) | 29.38  (± 9.4) |
| DI* | 0.077  (± 0.01) | 0.089  (± 0.01) | 0.33  (± 0.05) | 0.42  (± 0.02) |  |  |  |  |  |  |  |  |
| V_Dmin_ | 99.8 %  (± 0.1%) | 99.8 %  (± 0.2 %) | 99.5 %  (± 0.3 %) | 98.7 %  (± 0.1 %) |  |  |  |  |  |  |  |  |
| *Dose Inhomogeneity (DI) = (D_5_-D_95_)/D_mean_, D_5_, D_95_ and D_mean_  are defined in table 1 | | | | | | | | | | | | |

| Table 4. The DVHs quantitative comparison of M-DAT and SIMAT plans concerning organs at risk for 10 supine and 10 prone patients. | | | | | | | | |
| --- | --- | --- | --- | --- | --- | --- | --- | --- |
|  | RC | | | BL | | | LF | |
|  | SIMAT  mean  (± SD) | M-DAT | | SIMAT  mean  (± SD) | M-DAT | | SIMAT  mean  (± SD) | M-DAT  Supine  and  Prone  mean  (± SD) |
|  |  | Supine  mean  (± SD) | Prone  mean  (± SD) |  | Supine  mean  (± SD) | Prone  mean  (± SD) |  |  |
| D_15%_ (Gy) | 64.00  (± 12.3) | 58.00  (± 11.5) | 44.50  (± 10.2) | 49.00  (± 17.3.0) | 50.00  (± 15.2.5) | 54.50  (± 11.4) | 38.00  (± 16.6) | 37.10  (± 14.3) |
| D_25%_ (Gy) | 58.00  (± 5.0) | 43.80  (± 6.2) | 33.00  (± 8.2) | 37.50  (± 15.4) | 45.00  (± 12.2) | 47.00  (± 15.2) | 32.00  (± 15.0) | 32.80  (± 10.5) |
| D_35%_ (Gy) | 52.50  (± 6.4) | 35.00  (± 8.3) | 25.30  (± 6.4) | 28.50  (± 12.4) | 38.00  (± 10.8) | 39.50  (± 11.5) | 31.50  (± 19.4) | 32.00  (± 16.8) |
| D_50%_ (Gy) | 49.00  (± 7.0) | 26.80  (± 7.5) | 16.30  (± 5.6) | 18.00  (± 10.5) | 30.50  (± 14.6) | 33.50  (± 12.2) | 26.10  (± 8.5) | 26.10  (± 9.7) |
| V_50_ | 38.0 %  (± 5.2 %) | 20.0 %  (± 3.2 %) | 10.0 %  (± 5.3 %) | 23.0 %  (± 8.2 %) | 15.0 %  (± 5.4 %) | 18.0 %  (± 5.6 %) | 0.0 % | 0.0 % |
| V_60_ | 22.0 %  (± 9.2 %) | 13.0 %  (± 6.2 %) | 7.0 %  (± 5.2 %) | 5.0 %  (± 2.2 %) | 9.0 %  (± 5.2 %) | 9.0 %  (± 4.2 %) | 0.0 % | 0.0 % |
