# Supplementary material for: Forward‐planning intensity‐modulated radiotherapy technique for prostate cancer
Source: J Appl Clin Med Phys. 2007 Nov 5;8(4):114–28. doi: 10.1120/jacmp.v8i4.2488 (PMC5722620; doi:10.1120/jacmp.v8i4.2488)
Supplement: Supplementary file 2 — Supplementary Material Files [file ACM2-8-114-s002.doc]

*The point by point reply for the final version.*

*Page 4, para 3, line 8,  Should the underscore be removed from Arc_therapy?*

*Removed*

*Page 7, para 2, line 1,  Change “The resulted” to “The resulting”.*

*Changed*

*Page 15, para 2, line 1,  Change “volume that” to “volume that is”.*

*Changed*

*Page 15, para 2, line 2,  Do not begin sentence with “So that”.  Perhaps join with the previous sentence.*

*Replaced by Consequently,*

*Page 15, para 3, line 2.  Perhaps change to “the PSA level in their blood”.  Also change next sentence to “weaned off”.*

*Changed*

*Page 17, para 1, line 3,  Change to “obtained by a single”.*

*Changed*

*Page 17, para 1, line 7,  Change to “treatment of the prostate”*

*Changed*

*Page 17, para 1, line 11.  Do not begin sentence with “So that”.  Perhaps change this sentence to begin with “This technique may be investigated …”*

*Changed*

*I am looking forward to your last revision, and publication of your paper in JACMP.*

*I am sending you these comments directly to avoid  delays in publications due to issues with our website.*

*Regards,*

*Gino*
